# Severe Childhood Malaria Syndromes Defined by Plasma Proteome Profiles

**DOI:** 10.1371/journal.pone.0049778

**Published:** 2012-12-04

**Authors:** Florence Burté, Biobele J. Brown, Adebola E. Orimadegun, Wasiu A. Ajetunmobi, Francesca Battaglia, Barry K. Ely, Nathaniel K. Afolabi, Dimitrios Athanasakis, Francis Akinkunmi, Olayinka Kowobari, Samuel Omokhodion, Kikelomo Osinusi, Felix O. Akinbami, Wuraola A. Shokunbi, Olugbemiro Sodeinde, Delmiro Fernandez-Reyes

**Affiliations:** 1 Division of Parasitology, Medical Research Council National Institute for Medical Research, London, United Kingdom; 2 Department of Paediatrics, College of Medicine, University of Ibadan, University College Hospital, Ibadan, Nigeria; 3 Department of Haematology, College of Medicine, University of Ibadan, University College Hospital, Ibadan, Nigeria; 4 Childhood Malaria Research Group, University College Hospital, Ibadan, Nigeria; London School of Hygiene and Tropical Medicine, United Kingdom

## Abstract

**Background:**

Cerebral malaria (CM) and severe malarial anemia (SMA) are the most serious life-threatening clinical syndromes of *Plasmodium falciparum* infection in childhood. Therefore it is important to understand the pathology underlying the development of CM and SMA, as opposed to uncomplicated malaria (UM). Different host responses to infection are likely to be reflected in plasma proteome-patterns that associate with clinical status and therefore provide indicators of the pathogenesis of these syndromes.

**Methods and Findings:**

Plasma and comprehensive clinical data for discovery and validation cohorts were obtained as part of a prospective case-control study of severe childhood malaria at the main tertiary hospital of the city of Ibadan, an urban and densely populated holoendemic malaria area in Nigeria. A total of 946 children participated in this study. Plasma was subjected to high-throughput proteomic profiling. Statistical pattern-recognition methods were used to find proteome-patterns that defined disease groups. Plasma proteome-patterns accurately distinguished children with CM and with SMA from those with UM, and from healthy or severely ill malaria-negative children.

**Conclusions:**

We report that an accurate definition of the major childhood malaria syndromes can be achieved using plasma proteome-patterns. Our proteomic data can be exploited to understand the pathogenesis of the different childhood severe malaria syndromes.

## Introduction

Human malaria caused by *Plasmodium falciparum* has an estimated annual global disease burden of 300 million clinical episodes, leading to one million deaths[1]–[4]. Eighty-five per cent of the cases and 90% of the mortality occurs in sub-Saharan Africa, mostly amongst children [5], [6]. Recent reports point to a reduction of malaria cases in parts of Africa [7]. However, Nigeria, the most populous country of Africa, accounts for a quarter of the global cases and a third of the malaria-attributable childhood deaths [2], [8], [9].

Cerebral malaria (CM) and severe malarial anemia (SMA) are the major severe disease syndromes in African children with a high level of mortality in the under-five age group. The current WHO case definitions for severe malaria combine *P. falciparum* blood stage parasitemia with coma, severe anemia or respiratory distress [10], and it is well documented that there is significant overlap across these syndromes [11]. Despite the fact that these WHO case definitions are sensitive and useful for clinical diagnosis, the pathogenesis of severe disease is not well understood. One disadvantage of the WHO clinical definitions is that they lack the specificity required to carry out studies aimed at understanding the pathogenesis of clinically different forms of childhood malaria.

Previous studies have attempted to define malaria syndromes by studying plasma correlates of severity using reductionist approaches with variable success[12]–[14]. Small sample sizes, a lack of validation cohorts and a focus on a small selection of host plasma proteins have limited these studies. To overcome such limitations we use a systems approach to define the plasma proteome profile during malaria infection and identify distinctive patterns that are characteristic of different disease states. Contrary to other proteomic approaches, high-throughput plasma proteome profiling enables simultaneous analysis of a large number of samples. Therefore plasma proteome profiling allows the use of statistical pattern-recognition methods to discover and validate proteome-patterns that discriminate disease states.

**Table 1:** Characteristics of discovery and validation study groups.

	Discovery Cohort					Validation Cohort				
Clinical Group:	CM	SMA	UM	DC	CC	CM	SMA	UM	DC	CC
**N**	71	82	214	116	173	40	40	80	50	80
**Age** months Median (IQR)	41 (36)	31 (26)	42 (54)	54 (72)	72 (103)	46 (40)	28 (19)	41 (44)	27 (71)	96 (36)
**Sex Ratio** Female:Male	39∶32	38∶44	89∶125	55∶61	94∶79	17∶23	18∶22	33∶47	20∶30	39∶41
**PCV** % Mean (sd) Min - Max	27.5 (5.7) 20–40	13 (2.2)* 6–15	29.1 (5.9) 20–57	25 (10.4) 5–48	36.9 (5.2)** 25–54	27.2 (5.9) 30–37	12.9 (2.1)* 9–15	30.4 (5.5) 20–50	29.8 (7.8) 12–44	34.1 (3.8)** 22–41
**Parasite Density** log(MP/µL)Median (IQR)	4.1 3.11–4.77	4.38 3.28–4.70	4.55 3.54–4.75	N/A	N/A	4.41 3.29–4.78	3.91 3.05–4.64	4.59 3.31–4.76	N/A	N/A

CM = Cerebral Malaria; SMA = Severe Malarial Anemia; UM = Uncomplicated Malaria; DC = Disease Controls; CC = Community Controls. N = Number of Patients; IQR = Inter-quartile Range; sd = Standard Deviation; MP = Malaria Parasites. PCV = Packed Cell Volume.

* All clinical groups PCV significantly different to SMA (p<0.05).

** All clinical groups PCV significantly different to CC (p<0.05).

We hypothesized that the plasma proteome during malaria infection reflects the molecules that are modulated as the severe status is established. In the present study we show that distinctive plasma proteome-patterns distinguish the different severe presentations of *P. falciparum* childhood malaria from the uncomplicated cases and also from well or unwell children without malaria.

**Figure 1:**
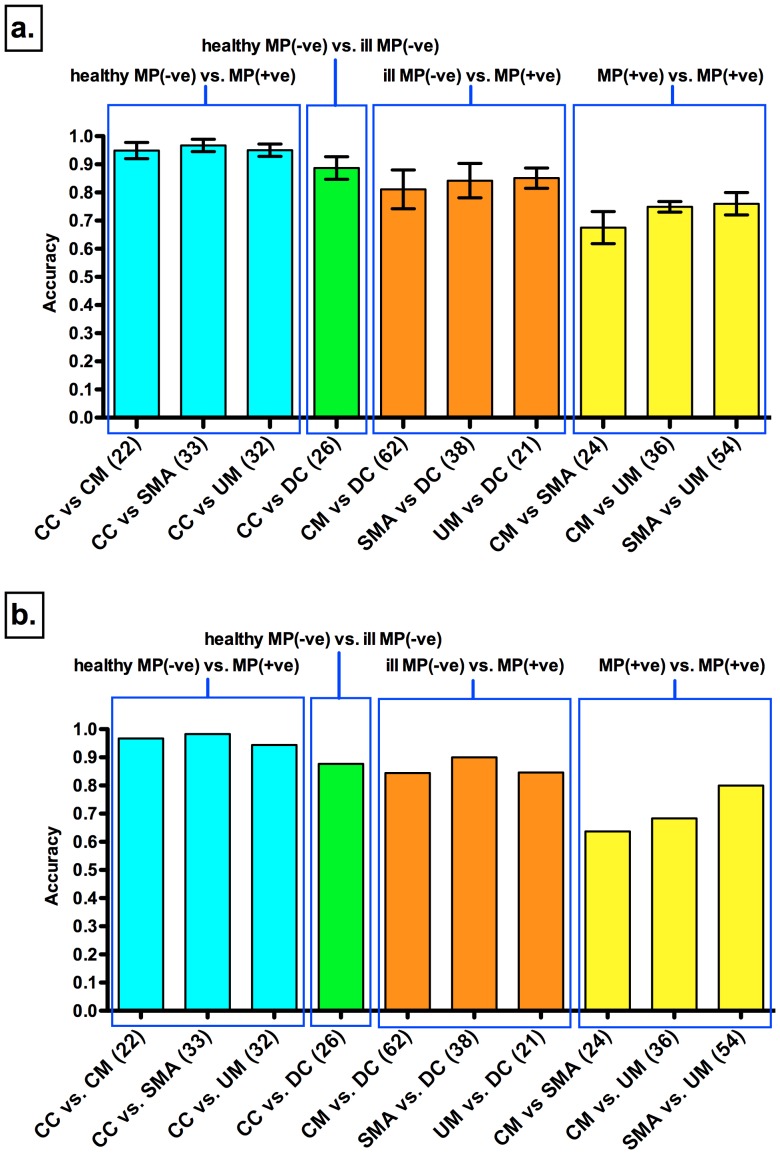
Discriminatory accuracy of proteome profiles across study groups. CM = Cerebral Malaria; SMA = Severe Malarial Anemia; UM = Uncomplicated Malaria; DC = Disease Controls; CC = Community Controls. Blue bars compare (CM, SMA and UM) with non-parasitemic CC. MP(−ve) = malaria parasite negative; MP(+ve) = malaria parasite positive. Green bar compares DC and CC (both non-parasitemic). Orange bars compare (CM, SMA and UM) with DC (non-parasitemic). Yellow bars compare CM, SMA and UM (all parasitemic) among themselves. In brackets are shown the number of relevant m/z clusters that make up the discriminatory proteome profile. (a.) Accuracy of predictive models built with relevant m/z clusters using the discovery cohort data. Error bars indicate +/− standard deviations obtained by 100 train/test randomizations of the data. (b.) Accuracy of the best predictive model from (a.) when applied to the validation cohort data.

## Methods

### Ethics Statement

Parents or guardians of study participants gave informed written consent. This research was approved by the joint ethics committee of the College of Medicine of the University of Ibadan and the University College Hospital Ibadan.

**Figure 2:**
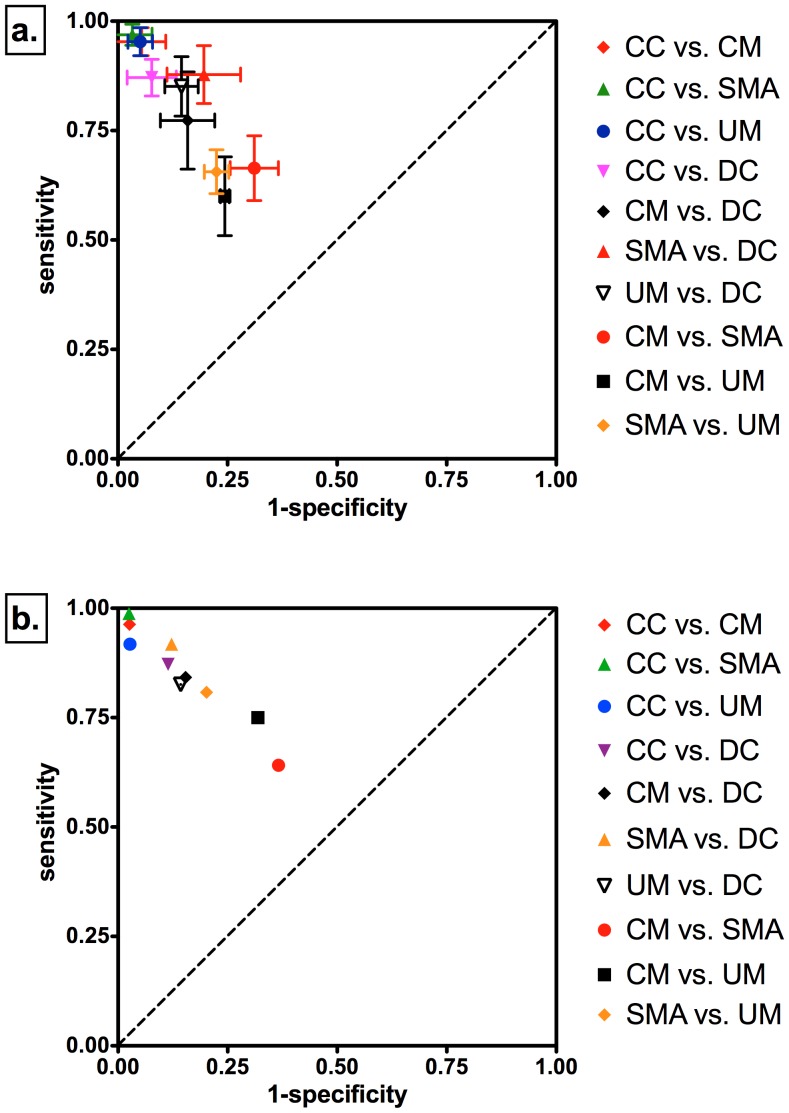
Discriminatory performance of proteome profiles across study groups in ROC space. CM = Cerebral Malaria; SMA = Severe Malarial Anemia; UM = Uncomplicated Malaria; DC = Disease Controls; CC = Community Controls. (a.) Discriminatory performance in ROC space of predictive models built with relevant m/z clusters using the discovery cohort data. Error bars indicate +/− standard deviations obtained by 100 train/test randomizations of the data. (b.) Discriminatory performance in ROC space of the best predictive model from (a.) when applied to the validation cohort data.

### Study Site

All study participants were recruited under the auspices of the Childhood Malaria Research Group (CMRG) at the 600-bed tertiary hospital University College Hospital (UCH) in the city of Ibadan, Nigeria in west sub-Saharan Africa. Ibadan is a densely populated urban setting with a population of 2.5 million inhabitants. Ibadan has a lengthy 8 months rainy season from March to October with malaria transmission and severe disease present all year round (holoendemic).

**Figure 3:**
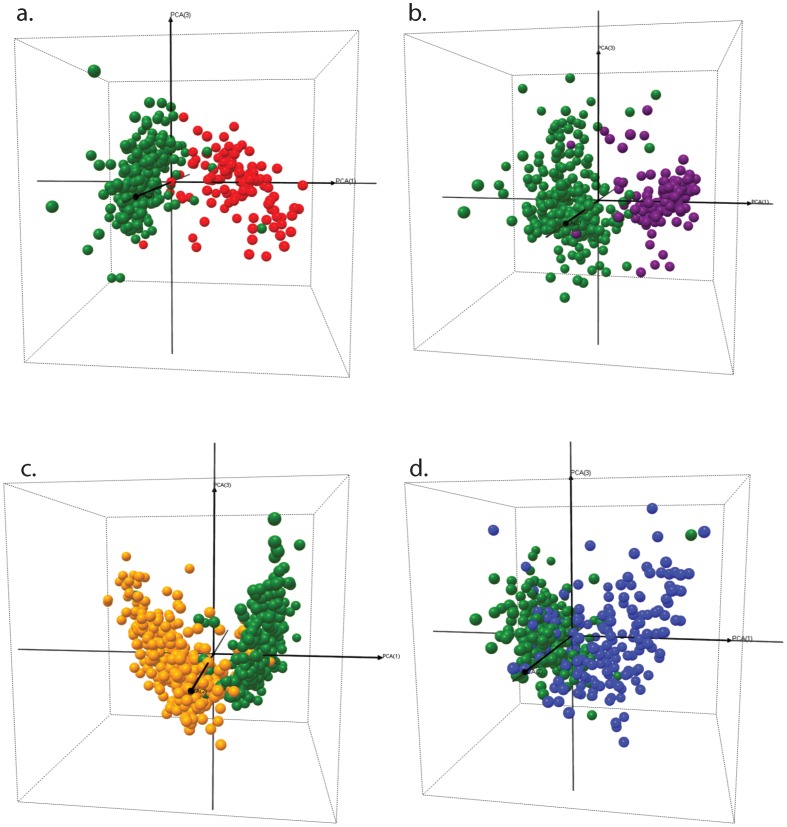
Visualization of community control (CC, non-parasitaemic) children *versus* other study groups. Each sphere represents an individual child proteome profile plotted in 3D space defined by the first three principal components. CM = Cerebral Malaria (red); SMA = Severe Malarial Anemia (purple); UM = Uncomplicated Malaria (yellow); DC = Disease Controls (blue); CC = Community Controls (green). (a.) CC vs. CM; (b.) CC vs. SMA; (c.) CC vs UM and (d.) CC vs. DC.

The study site is located in the UCH Ibadan Department of Paediatrics. We screen about 12,000 children attending the hospital (ill and well) for malaria parasites per year. Our studies report 11.3% SMA and 19.7% CM admissions in the parasitized children under five years of age [9].

**Figure 4:**
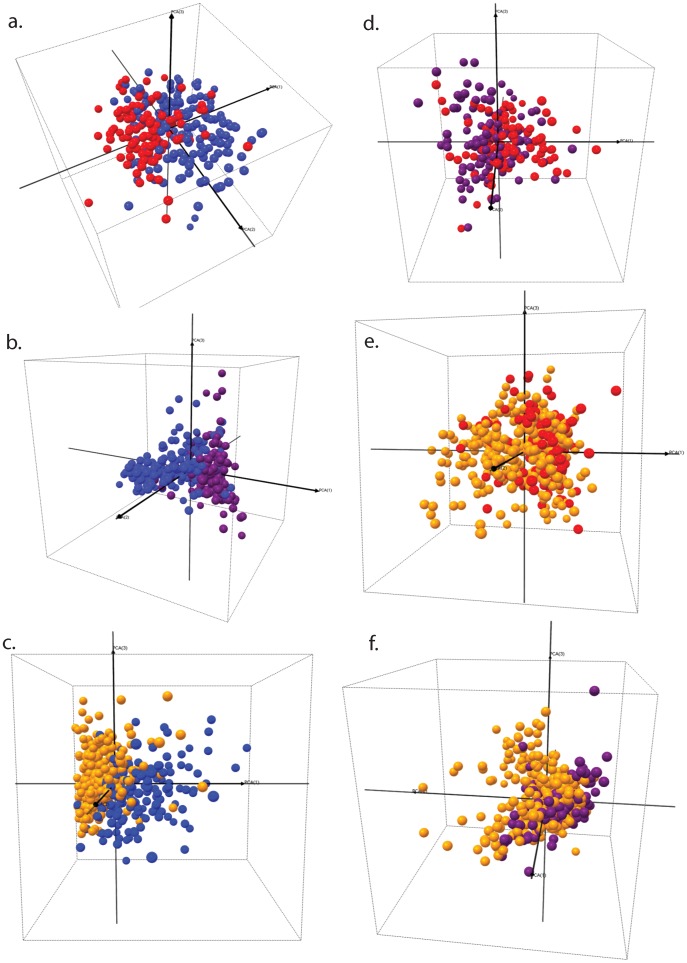
Visualization of (a-c) disease control (DC, non-parasitemic) children *versus* parasitemic children groups; (d-f) among CM, SMA and UM parasitaemic children groups. Each sphere represents an individual child proteome profile plotted in 3D space defined by the first three principal components. CM = Cerebral Malaria (red); SMA = Severe Malarial Anemia (purple); UM = Uncomplicated Malaria (yellow); DC = Disease Controls (blue). (a.) DC vs. CM; (b.) DC vs. SMA; (c.) DC vs. UM; (d.) CM vs. SMA; (e.) CM vs. UM and (f.) SMA vs. UM.

### Study Design and Case Definitions

The participants in this study were recruited during 2006 to 2009 as part of a larger prospective case-control study of childhood severe malaria currently ongoing under the auspices of the CMRG. This case-control study was divided into a Discovery Cohort consisting of those patients recruited during 2006 to 2008 and a Validation Cohort made up of those recruited in the 2008 to 2009 period.

**Figure 5:**
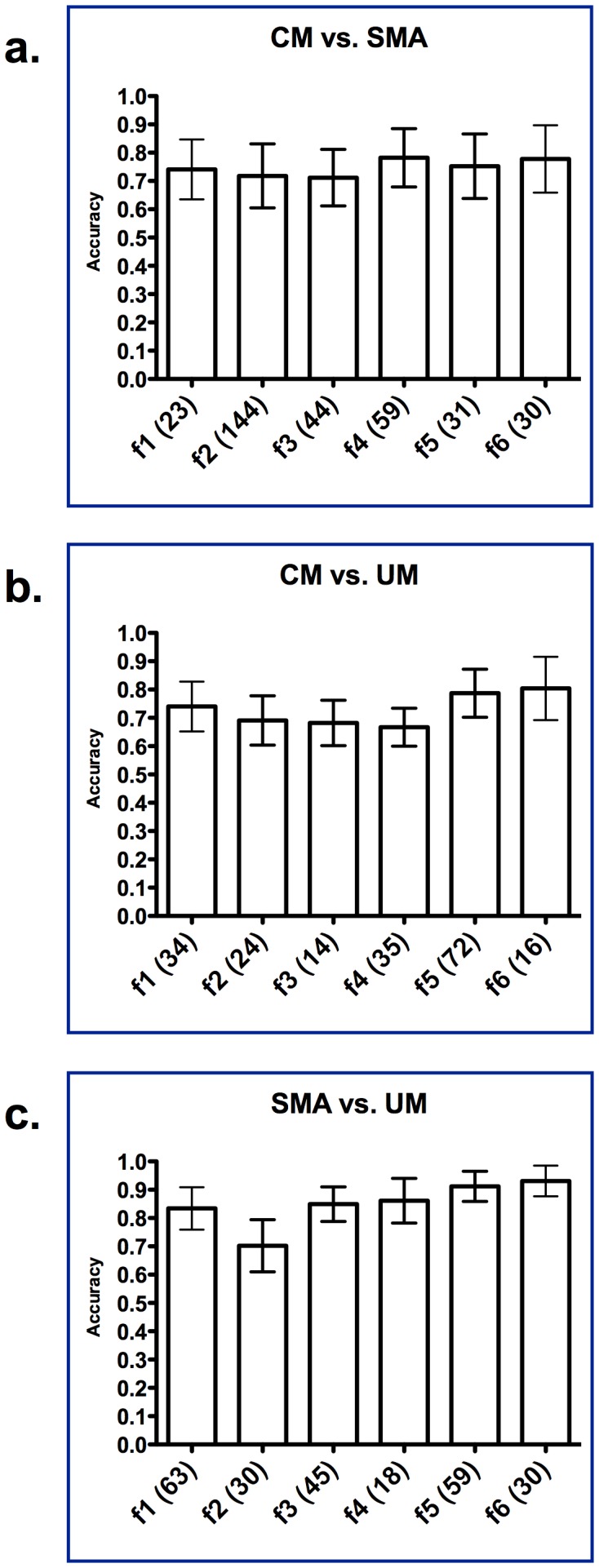
Discriminatory accuracy of proteome profiles across six anionic plasma fractions (f1 to f6) among CM, SMA and UM (all parasitemic) groups of children. CM = Cerebral Malaria; SMA = Severe Malarial Anemia; UM = Uncomplicated Malaria. f1 to f6 represent anionic plasma fractions at pH 9.0 (f1), pH 7.0 (f2), pH 5.0 (f3), pH 4.0 (f4), pH 3.0 (f5) and organic phase (f6). In brackets are shown the number of relevant m/z clusters that make up the discriminatory proteome profile. (a.) CM vs. SMA; (b.) CM vs. UM; (c.) SMA vs. UM.

Malaria parasites were detected and counted by microscopy following Giemsa staining of thick and thin blood films [15]. Children with severe malaria were recruited on admission from the Otunba Tunwase Children’s Emergency Ward (OTCHEW). Children with uncomplicated malaria were recruited as part of a daily routine malaria parasite screening at the Children’s Out-patient Clinics (CHOP). Malaria-negative ill children were recruited either at admission from OTCHEW or from the Department of Paediatrics In-patient wards. Malaria-negative healthy community control children were recruited from local vaccination clinics as well as during school visits across several Ibadan districts.

**Figure 6:**
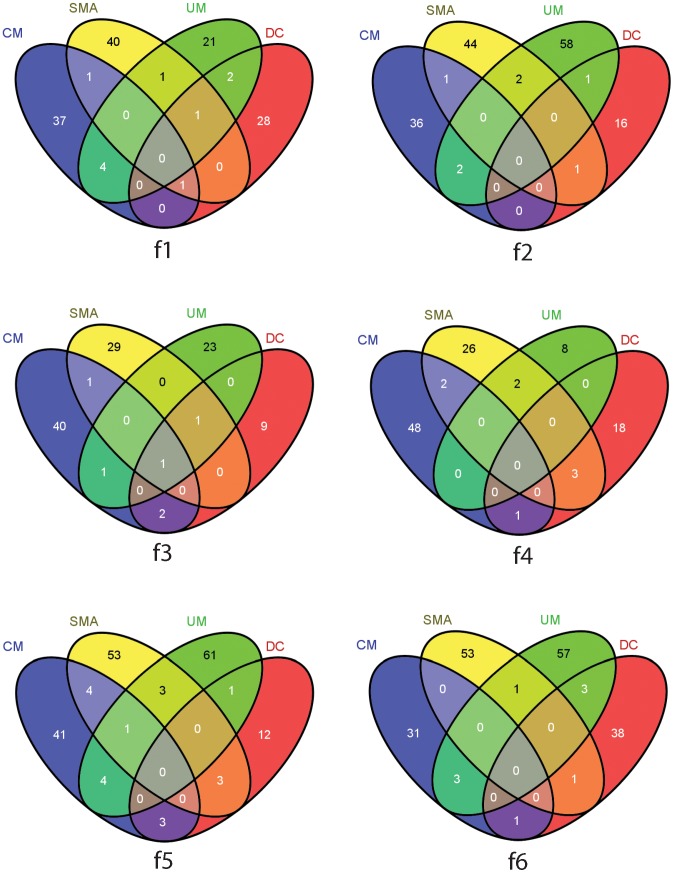
Proteome-pattern overlap. The Venn diagram represents the level of overlap between the patterns that individually discriminate CM, SMA, UM and DC from CC. CM = Cerebral Malaria; SMA = Severe Malarial Anemia; UM = Uncomplicated Malaria. f1 to f6 represent anionic plasma fractions at pH 9.0 (f1), pH 7.0 (f2), pH 5.0 (f3), pH 4.0 (f4), pH 3.0 (f5) and organic phase (f6).

We recruited children aged from 6 months to 13 years using five participant definitions. The malaria-positive children, the cases, are Uncomplicated Malaria (UM), Severe Malarial Anemia (SMA) and Cerebral Malaria (CM). The malaria-negative children, the controls, are Disease Control (DC) and Community Controls (CC). We followed the WHO criteria for severe *P. falciparum* malaria [10]. Cerebral malaria cases were defined as children in unrousable coma for at least one hour in the presence of asexual *P. falciparum* parasitemia with normal cerebrospinal fluid. A Blantyre coma score less than 2 was used to assess coma status. Children with hypoglycemia were excluded from the study. Added to the strict clinical and laboratory definitions of CM, our study patients recover consciousness after effective antimalarial therapy. We excluded from this study those CM patients who died. Our overall mortality rate for CM is of the order of 10%. Severe malarial anemia cases were defined as conscious children with Packed Cell Volume (PCV) less than 16% in the presence of *P. falciparum* parasitemia. We excluded from this study those SMA patients who died. Our overall mortality rate for SMA is less than 1%. Uncomplicated malaria cases were defined as febrile children with *P. falciparum* parasitemia who did not require hospital admission. Our study was designed to discover and validate plasma proteome changes in dichotomous cases for which we only included those children with CM and UM with PCV greater than 20% (Table 1). We excluded from the study blood culture positive cases. Although we did not carry out blood cultures in all severe malaria patients, the cases recruited into this study are those in whom septicemia was not suspected and who were successfully treated with antimalarial alone.

The DC group consists of malaria-negative children with infectious diseases such as meningitis, otitis media, diarrhea and upper respiratory tract infections. It also includes mild to moderately anemic children and children admitted for surgery.

### Clinical Data and Sample Collection

Participants’s clinical data were collected using a malaria-tailored questionnaire designed by the CMRG. A 2.5 ml blood sample was obtained from each participant in an EDTA blood collection tube for subsequent plasma separation. Blood samples were kept on ice and transferred to the central malaria laboratory. Plasma for this study was harvested by centrifugation (1000 *g*, 10 minutes), aliquoted and frozen at −80°C no later than 4 hours following collection.

### Clinical Laboratory Analysis

Packed cell volume (PCV) was measured using the microhaematocrit method [15]. Briefly, Blood was obtained in capillary tubes. Tubes were centrifuged at 12,000 g for 5 minutes. The percentage cell volume compared to the whole tube volume was calculated (i.e. PCV). Mean (± standard deviation, sd), minimum and maximum PCV for each clinical group are tabulated in Table 1. For discovery and validation cohort, these data were compared using a one-way multiple ANOVA test (p<0.05).

Malaria parasites were detected and counted by microscopy following Giemsa staining of thick and thin blood films [15]. Malaria Parasite (MP) densities were calculated as follows MP/µL = [(number malaria parasites/wbc) x 8,000] and expressed as log(MP/µL) for each malaria-positive clinical group (Table 1). The microscopic criterion for declaring a participant to be free of malaria was the absence of parasites in 100 high-power (1000X) fields. One in 10 thick blood films were randomly selected and independently reviewed by local experienced microscopists not part of the research team.

### Solid-phase High-throughput Plasma Fractionation and Proteomic Profiling

Crude plasma was profiled using Surface Enhanced Laser Desorption/Ionization-Time Of Flight (SELDI-TOF) mass spectrometry. All plasma samples underwent two freeze-thaw cycles prior to analysis. Plasma samples were coded, blinded and randomized before application onto the following solid-phase fractionation surfaces (ProteinChip® arrays Bio-Rad): weak-cation exchange (CM10), strong-anion exchange (Q10) and reverse phase (H50) as previously described [16]. Liquid handling steps were automated using a Biomek 3000 Laboratory Automation Workstation (Beckman Coulter) and a 96 well Bioprocessor® (Bio-Rad). Each ProteinChip® 96 well Bioprocessor® included 1 quality control plasma standard derived from a single healthy individual, placed at random. Mass spectra were generated on a System 4000 Bio-Rad ProteinChip® mass spectrometer. Spectral peaks corresponding to mass/charge (*m/z*) clusters were detected and clustered using ProteinChip® Datamanager Client 4.1 software (BioRad). Mass spectrometer calibration was performed using All-in-1 Peptide and Protein calibrants (Bio-Rad). Reproducibility was determined by measuring the inter-ProteinChip® coefficient of variation (CV) for the quality control spectra, based on all peaks in the spectrum with intensity >1 µA. Overall interchip CV for the quality control sample was 20%, consistent with similar studies.

### Liquid-phase High-throughput Anionic Exchange Plasma Fractionation

Liquid-phase anion-exchange fractionation of plasma samples was carried out using the ProteinChip® Fractionation Kit (Bio-Rad) according to the manufacturer’s instructions with a Biomek 3000 Laboratory Automation Workstation. Six fractions were obtained from each sample eluting at pH 9.0 (f1), pH 7.0 (f2), pH 5.0 (f3), pH 4.0 (f4), pH 3.0 (f5) and organic phase (f6).

### Data Analysis

We selected subsets of the most relevant mass clusters in the discovery cohort groups using the weighted Kernel-based Iterative Estimation of Relevance Algorithm [17] (wKIERA) that combines a stochastic-search estimation of distribution algorithm with a kernel pattern-recognition method. We then used discovered relevant subsets of mass clusters to build discriminatory predictive models. We adopted a supervised learning approach to derive a classification rule using the Support Vector Machine (SVM) method [18]. Briefly, we used 10-fold cross validation to select parameters for the SVM. For the final model parameters, we selected those that gave the overall highest accuracy across the whole 10-fold cross validation. To obtain robust accuracy estimates for the classifier on the discovery data, we took 100 random re-samplings of the data, using 80% for training and 20% for testing. We selected as a final classifier the one that produced the highest accuracy and was then tested on the validation cohort data. Results were expressed as sensitivity, specificity and accuracy (proportion of correct classifications) and plotted on Receiver Operator Characteristic (ROC) space plots.

Our multivariate statistical tests included testing against age or sex to ascertain that significant pattern changes in the proteome were not dependent on those variables in the population studied.

To visualize the covariance within the mass spectral profiles we used Principal Component Analysis (PCA). PCA encapsulates the covariance within a set of variables by extracting a ranked set of independent factors or principal components. The first 3 components encompass a high proportion (∼95%) of the informational content of a multivariate dataset. We plotted each patient with respect to the first 3 components, in 3-dimensional space, color-coding according to patient group.

## Results

### Study Participants

A total of 946 children participated in this study as part of the discovery and validation case-controlled cohorts. The discovery cohort comprised of 367 malaria-positive children with either Cerebral Malaria (CM), Severe Malarial Anemia (SMA) or Uncomplicated Malaria (UM), and 289 malaria-negative children who were either Disease Controls (DC) or Community Controls (CC) (Table 1). The validation cohort was prospectively recruited after the discovery cohort and comprised 160 malaria-positive children with either CM, SMA or UM, and 130 malaria-negative DC or CC children (Table 1). PCV and malaria parasite (MP) densities are presented in Table 1. Consistent with the recruitment criteria, both discovery and validation SMA groups had PCVs below 16% (Table 1). There was mild anemia across CM, UM and DC groups in both cohorts, whereas CC had normal mean hematocrit (Table 1). Parasite densities across all the infected groups were similar (Table 1).

### Plasma Proteome-patterns Define the Major childhood Malaria Syndromes

To compare the proteome-patterns of the study groups, we fractionated plasma samples by three different chromatography procedures on solid-phase surfaces (weak-cationic and strong-anionic ion-exchange, and reverse-phase) followed by Time-Of-Flight mass spectrometry. The resulting mass spectra from each of the surfaces contained a series of mass/charge ratio (m/z) peak clusters, each representing a protein of a particular mass. A set of proteins that are present, absent or at a different level in the samples defines a proteome-pattern that may discriminate between two or more of the study groups. To discover such patterns we applied statistical pattern recognition algorithms to the profiles and the selected number of discriminating proteins for each of the pairwise group comparisons is shown in Figure 1, as the numbers in parentheses (Data S1). We built predictive models with the selected proteome-pattern for each study group comparison using a non-parametric supervised learning statistical framework. The discriminatory accuracy of these predictive models in the discovery cohort groups is shown in Figure 1a. To determine differences for malaria-positive children from healthy malaria-negative children we compared individually the plasma proteome of CM, SMA and UM groups with that of the CC group.

Overall, 22 to 33 proteins composed the discriminatory patterns with accuracies above 90% across the three comparisons (Figure 1a, blue bars). Twenty-six proteins discriminated healthy from ill (hospital admitted) malaria-negative children (CC vs. DC) with similar accuracy (Figure 1a, green bar). To examine proteins that are specific to malaria infection we compared each of the malaria-positive groups (CM, SMA, UM) to the DC group, obtaining discrimination accuracies above 80% (Figure 1a. orange bars). Finally, to assess differences between defined malaria syndromes we compared the malaria-positive groups (Figure 1a. yellow bars). In the comparison between CM and SMA, the two major severe syndromes, the accuracy was 70% (24 proteins). Higher accuracies between 70 to 80% were observed when samples from either CM or SMA groups were compared to UM children, using 36 and 54 proteins, respectively.

To validate the accuracy of the discrimination for the discovered plasma proteome-patterns, we tested the predictive models on the validation cohort groups (Figure 1b). The best predictive model for each group comparison in the discovery cohort was asked to predict the group class in the validation cohort. We found that the predictive models obtained using the discovery cohort had similar accuracy for discrimination in the different group comparisons for the validation cohort (Figure 1b). We compared the sensitivity and specificity of the predictive models for both discovery and validation cohort groups in ROC space and found them to be similar (Figure 2).

We then used Principal Component Analysis (PCA) on the selected proteins to visualize the separation of patient groups. The CC group clustered tightly together (Figure 3, green spheres). Individual malaria-positive groups showed good separation from the malaria-negative CC group (Figure 3a–c) indicating that regardless of disease severity there are significant differences in the proteomes of the groups. The heterogeneous DC group had a more dispersed cluster pattern with little overlap with the CC group (Figure 3d). The DC group, despite being distinct, showed different degrees of overlap with the malaria-positive groups (Figure 4a–c). Of these comparisons, the CM vs. DC patient groups showed the greatest level of cluster dispersion (Figure 4a) indicating greater covariance in the proteins that define these groups. We then compared the malaria-positive patient groups among themselves (Figure 4d–f). CM and SMA groups showed overlap at the cluster interface and clearer segregation at the periphery; in the comparison of both severe forms (CM and SMA) with UM we observed that the severe patient groups had compact center clusters surrounded by a more disperse cluster of the UM patient group.

### Reduction of Plasma Proteome Complexity Provides Further Discriminatory Proteins for Severe Childhood Malaria

We simplified further the complexity of the plasma proteome by high-throughput liquid-phase anion-exchange fractionation followed by solid-phase weak-cation exchange fractionation prior to protein mass determination in the spectrometer on a subset of the samples. We assessed the discriminatory accuracy of relevant proteins obtained from each of the six anion-exchange fractions (Figure 5, f1 to f6) (Data S2). The reduction in the complexity of each fraction of the plasma samples resulted in a larger subset of proteins that improved discrimination between the malaria syndromes. Sets of proteins that distinguish between SMA and CM groups (Figure 5a, f1 to f6 in brackets) slightly outperformed the proteome-pattern from non-fractionated plasma. Sets of proteins differentiated the CM and UM groups with accuracies ranging from 70 to 80% (Figure 5b, f1 to f6 in brackets) and distinguished between SMA and UM with comparable accuracy (Figure 5c, f1 to f6 in brackets).

We carried out an overall analysis of plasma proteome pattern overlap by comparing the discovered sets of proteins that discriminate UM, CM, SMA (malaria-positive) and DC (malaria-negative) ill children from the malaria-negative well children CC (Figure 6, f1 to f6). We show that each plasma fraction (f1 to f6) contains a set of proteins that clearly define both the malaria-positive and malaria-negative ill children to those malaria-negative well children in the community. Furthermore, we also show that the set of proteins that discriminate SMA and CM from UM have very little overlap across the six plasma fractions (Figure 6, f1 to f6).

## Discussion

In the present study we carried out a large case-control study of severe childhood malaria, using a discovery cohort to define discriminatory plasma proteome-patterns and a second cohort to validate our findings, at the main tertiary hospital of the city of Ibadan, Nigeria.

We show that proteome-patterns from both crude and pre-fractionated plasma samples accurately define childhood malaria syndromes in the discovery cohort. We confirmed these findings using a prospectively collected validation cohort. Malaria infection introduces distinguishable changes in the plasma proteome of children as seen by the striking differences between the malaria-negative CC and the malaria-positive children groups. The plasma proteome differences are specific for the malaria disease process and not surrogate markers of acute illness, as we are able to accurately distinguish between malaria-negative ill children and malaria-positive groups independently of their disease severity. We have also discovered plasma proteome differences that are specific to each of the childhood malaria syndromes assessed in the present study. Our findings provide a starting point to refine the current WHO definitions of these syndromes, which lack the necessary specificity to further study severe malaria pathogenesis.

We show that assessing the plasma proteome of the major malaria syndromes provides an unbiased discovery of combination of proteins that could be used to deepen our understanding of the pathogenesis of childhood malaria. This is supported by the finding that we can discriminate children with uncomplicated malaria from those with severe malarial anemia or cerebral malaria in both discovery and validation cohorts. These proteome-patterns encapsulate what changes differentiate uncomplicated malaria from the severe cases.

Overall, accuracy of discrimination between the CM and SMA was lower than that in the comparison of each of these syndromes with the UM group. The degree of overlap between CM and SMA goes beyond that expected from strict application of the WHO case definitions used in this study. Nevertheless, the plasma proteome-pattern discriminated with over 70% accuracy between the severe groups. This suggests that beyond common underlying mechanisms, such as acute inflammation, there are significant differences in the pathogenesis of the severe syndromes studied.

Our large cohorts allowed us to statistically validate the pattern-based proteome definitions of the major childhood malaria syndromes. Although the mass spectrometry platform used in our study does not provide direct molecular identification, the chromatographic chemistry used and the mass-to-charge (*m/z*) ratio can be exploited to guide the identification of the set of proteins relevant for discrimination between syndromes. Plasma proteome profiling has been used to define a variety of disease states[16], [19]–[24] as there is growing recognition of the advantages of using ‘omics’-based methods to achieve sufficient levels of accuracy [24]. Our study showed that complex plasma protein patterns were necessary to discriminate between the different malaria syndromes. This further underlines the advantage of using unbiased high-throughput pattern recognition based methods.

In many infectious diseases, there are clinically important distinctions to be made between different manifestations associated with the same underlying pathogen and malaria clinical syndromes are a clear case in point. The pathogenesis of malaria due to its erythrocytic cycle occurs in the cardiovascular system and it is plausible that proteome changes in organs such as brain, spleen, kidney and bone marrow can be reflected in the plasma proteome. Our study confirms that there are proteome changes characteristic of the clinical malarial syndromes with different level of accuracy. Furthermore, host modulation by the pathogen is likely to generate changing patterns of protein expression associated with the progression of severe malaria syndromes and our current studies are designed to address such changes.

The lack of specific childhood malaria definitions has limited the progress on understanding the pathology of the major severe syndromes. To the best of our knowledge this study is the first to show that a panel of proteins, defined as a proteome-pattern, dissects clinical malaria syndromes. Further identification of the proteins that comprise the proteome-patterns will provide hints to the underlying pathogenesis on each of the syndromes. Furthermore, these proteome-patterns provide a reference point to facilitate the identification of other complex and overlapping severe childhood malaria syndromes.

## Supporting Information

Data S1 Solid-phase fractionation data.
(XLS)

Data S2 Liquid-phase fractionation data.
(XLS)
